# The incidence of nosocomial bloodstream infection and urinary tract infection in Australian hospitals before and during the COVID-19 pandemic: an interrupted time series study

**DOI:** 10.1186/s13756-023-01268-2

**Published:** 2023-07-03

**Authors:** Brett G Mitchell, Andrew J Stewardson, Lucille Kerr, John K Ferguson, Stephanie Curtis, Ljoudmila Busija, Michael J Lydeamore, Kirsty Graham, Philip L Russo

**Affiliations:** 1 School of Nursing, Avondale University, Cooranbong, NSW 2265 Australia; 2 Nursing and Midwifery, Monash University, Frankston, VIC 3199 Australia; 3 Gosford Hospital, Central Coast Local Health District, NSW, 2250 Australia; 4 Department of Infectious Diseases, The Alfred and Central Clinical School, Monash University, Melbourne, VIC 3004 Australia; 5 Department of Nursing Research, Cabrini Institute, Malvern, VIC 3144 Australia; 6 School of Nursing and Midwifery, Deakin University, Burwood, Australia; 7 Division of Medicine, John Hunter Hospital, Newcastle Regional Mail Centre, 2310 NSW, Australia; 8 University of Newcastle, Callaghan, NSW 2308 Australia; 9 Infection Prevention Service, Hunter New England Health, John Hunter Hospital, NSW, 2310 Australia; 10 Department of Epidemiology and Preventive Medicine, School of Public Health and Preventive Medicine, Monash University, Melbourne, VIC 3004 Australia; 11 Department of Econometrics and Business Statistics, Monash University, Melbourne, 3800 Australia; 12 Infection Prevention and Control, Central Coast Local Health District, Gosford, NSW 2250 Australia

**Keywords:** Infection prevention, COVID-19, Surveillance, Healthcare associated infection, Blood cultures, Urine cultures

## Abstract

**Background:**

The COVID-19 pandemic has had a significant impact on healthcare including increased awareness of infection prevention and control (IPC). The aim of this study was to explore if the heightened awareness of IPC measures implemented in response to the pandemic influenced the rates of healthcare associated infections (HAI) using positive bloodstream and urine cultures as a proxy measure.

**Methods:**

A 3 year retrospective review of laboratory data from 5 hospitals (4 acute public, 1 private) from two states in Australia was undertaken. Monthly positive bloodstream culture data and urinary culture data were collected from January 2017 to March 2021. Occupied bed days (OBDs) were used to generate monthly HAI incidence per 10,000 OBDs. An interrupted time series analysis was undertaken to compare incidence pre and post February 2020 (the pre COVID-19 cohort and the COVID-19 cohort respectively). A HAI was assumed if positive cultures were obtained 48 h after admission and met other criteria.

**Results:**

A total of 1,988 bloodstream and 7,697 urine positive cultures were identified. The unadjusted incident rate was 25.5 /10,000 OBDs in the pre-COVID-19 cohort, and 25.1/10,000 OBDs in the COVID-19 cohort. The overall rate of HAI aggregated for all sites did not differ significantly between the two periods. The two hospitals in one state which experienced an earlier and larger outbreak demonstrated a significant downward trend in the COVID-19 cohort (p = 0.011).

**Conclusion:**

These mixed findings reflect the uncertainty of the effect the pandemic has had on HAI’s. Factors to consider in this analysis include local epidemiology, differences between public and private sector facilities, changes in patient populations and profiles between hospitals, and timing of enhanced IPC interventions. Future studies which factor in these differences may provide further insight on the effect of COVID-19 on HAIs.

**Supplementary Information:**

The online version contains supplementary material available at 10.1186/s13756-023-01268-2.

## Background

The COVID-19 pandemic has seen an unprecedented increase in awareness and focus on infection prevention precautions, including hand-hygiene, cleaning, air quality, ventilation and correct use of personal protective equipment (PPE) [1]. Recent research has documented that although standard precautions were adopted globally prior to the pandemic, deficits in implementation and compliance persist [2–4]. With an increased focus on infection prevention and control practices and processes in healthcare settings as a result of the pandemic, it could be hypothesised that this in turn may have a positive effect on reducing the overall risk of infection transmission in these settings. Conversely, hospitals and healthcare workers have been under enormous strain from COVID-19 and this may result in a reduced focus on preventing infections other than COVID-19.

Emerging research contains mixed results about the effect the COVID-19 pandemic has had on the rates of healthcare associated infections (HAIs). Substantial increases in central-line associated bloodstream infections and catheter associated urinary tract infections have been observed, along with an increase in contaminated specimens and potential reduction in local HAI reporting [5–7]. Researchers have suggested that this may be due to: resources shortages; influx of patients; changing recommendations; and general stress [5–7]. However, reductions in *Clostridioides difficile* have also been reported, [1, 8, 9] and generally a lower rate of multidrug resistant organisms - although this was in an area which was at the time not significantly affected by COVID-19 infections [10] These have been attributed to the increased awareness and practice of standard precautions [1, 10, 11].

The infection prevention challenges presented by COVID-19 are significant. To prepare for the admission and treatment of COVID-19 positive patients, a number of new and modified infection prevention initiatives have been implemented across healthcare sites. These include, but are not limited to: an overall heightened awareness of infection prevention; increase in education regarding PPE; increase in the use of PPE; increase in promotion of hand hygiene; changes to cleaning regimes; restriction in visiting hours; improvement in ventilation and limited patient movement [12]. Whilst the correct and appropriate use of PPE, adequate air quality, hand hygiene and cleaning are fundamental in every infection prevention program, the heightened awareness COVID-19 has introduced, may mean there is increased compliance and diligence. At the same time, whilst preventing the spread of COVID-19, these activities will also prevent many other types of infection. On the other hand, as emerging research is indicating, the increased stress on healthcare workers and organisations may increase HAIs, [13] particularly given evidence that increased glove use often leads to poor hand hygiene compliance [14]. There are also several reports of increases in carbapenemase-producing Enterobacteriaceae in intensive care units in the context of COVID-19 and increased infection prevention activity related to non compliance with PPE, misuse of gloves, high antibiotic use and overwork [15–18]. The overall aim of this study is to explore if there has been any effect on HAI rates as a result of the increased infection prevention awareness brought about by COVID-19.

## Methods

### Study design

The study was a three-year retrospective review of inpatient laboratory data.

### Setting and population

Data were sourced from five Australian hospitals from two different Australian jurisdictions (New South Wales [NSW] and Victoria). These five hospitals consisted of four acute public hospitals (two Principal Referral Hospitals [Hospitals A and B]), and two Acute Group A hospitals [Hospitals C and D]) and one acute private hospital (Private Acute Group A [Hospital E]). Differences between these hospital types are detailed in Supplementary Table S1. Combined, these hospitals have over 2400 overnight beds and over 290,000 hospital admissions per year.

We constructed two cohorts; first, the pre-COVID-19 cohort, defined as inpatients who had specimens collected between January 2017 to February 2020, and second, the COVID-19 cohort, defined as inpatients who had specimens collected between March 2020 to March 2021, inclusive. This time point was chosen following the first identification of a COVID-19 case in Australia on 25th January 2020.

### Data sources

Microbiology data were obtained from the laboratories of participating hospitals for the period of January 2017 through to March 2021 (inclusive) for positive bloodstream and urine cultures. For each positive culture, patient level data were collected, including age, gender, date of admission, date of specimen collection and name of organism. Positive cultures that were collected within 48 h of admission, and repeat bloodstream cultures within 14 days, or urine cultures within 30 days, were excluded. To generate the incidence rate, monthly occupied bed day (OBD) [19] data were collected from each hospital for the same time period. To allow for uniform reporting of organisms, each organism reported from the source was categorised into a pathogen group (Supplementary Table S2).

### Definitions

For the purposes of this study, we applied the following definitions for HAIs:

- Bloodstream infection (BSI): positive culture collected > 48 h post admission.
- Urinary tract infection (UTI): positive culture collected > 48 h post admission.

### Statistical analyses

Interrupted time series (ITS) regression analyses with Newey-West autocorrelated errors [20] were carried out to assess differences in the log-transformed level and trend of HAI between the pre-COVID-19 and COVID-19 periods. The regression is performed on log-transformed data as the outcome variable (cases per 100,000 OBD) cannot be less than zero. The transformation ensures that the predicted outcome variable will remain non-negative. The ITS models assessed the baseline rate of HAI (intercept), trend during the pre-COVID-19 interval (slope), and the change in slope between the two time periods. Values with a response variable of zero had a small pseudo-count added to ensure the transformation was valid. Prior to analyses, model assumptions were evaluated through the inspection of autocorrelations and model residuals. Infection rates were also examined for potential seasonal trends, with no discernible seasonal trends detected. To ensure that the models accounted for the correct autocorrelation structure, Baum and Schaffer autocorrelation test for autocorrelation was used to test for up to 12 lags. Lags that had significant autocorrelations were incorporated into the model [21]. In all statistical analyses, nominal alpha level of 0.05 was used to interpret the results of significance tests.

To create the time series, the number of infections and number of OBD were aggregated by month. HAI rates were calculated as a ratio of the number of infections (numerator) in a given month to the corresponding number of OBD (denominator) and expressed as a rate per 10,000 admissions. We assessed changes in HAI rates overall, aggregated across hospitals, as well as changes in HAI rates for each hospital. We also assessed changes in the rates of BSI and UTI pooled across all sites and separately for each site. To assess the influence of individual sites on the overall HAI rates, jack-knife sensitivity analyses were undertaken by removing one hospital at a time and estimating ITS model for the rates pooled across the remaining hospitals.

## Results

Positive culture data from all hospitals were collected on specimens taken between 1 and 2017 to 31 March 2021. All hospitals reported data on BSI and UTI.

A total of 9,685 positive cultures (1,988 bloodstream and 7,697 urine) from 8,194 patients were included in the final analysis. The median age of the pre-COVID-19 cohort was 71 (quartile range 58–82) and 59% (3843/6481) were female. In the COVID-19 cohort the median age was 71 (quartile range 59–82) and 58% (992/1713) were female. The mean monthly number of occupied bed days combined in the pre COVID-19 cohort was 75,317 compared to 73,157 for the COVID-19 cohort. All sites reported a notable drop in occupied bed days in April 2020, but by June 2020 numbers had returned to similar pre COVID-19 numbers (Supplementary Figure S1).

Hospital A contributed the most culture positive episodes with 4,792, followed by Hospital B 2,943 episodes, Hospital E 1,614 episodes, Hospital C 230 episodes and Hospital D 106 episodes. The unadjusted incidence rates for all HAIs in the pre-COVID-19 cohort was 25.5 per 10,000 OBDs (95%CI:24.9–26.1) and in the COVID-19 cohort was 25.1 per 10,000 OBDs (95%CI:24.1–26.1). (Table 1) Sensitivity analysis on the influence of each site on combined BSI and UTI infections demonstrated that hospital A had a significant downward influence in the pre-COVID-19 cohort (p = 0.008), and Hospitals B and E had a significant upward influence on the COVID-19 cohort (p = 0.009 and p < 0.001 respectively. (Supplementary Figure S2).

**Table 1:** Unadjusted incidence rates per 10,000 occupied bed days (OBDs)

	Pre-COVID-19 cohort (Jan 2017 – Feb 2020)		COVID-19 cohort (Mar 2020 – Mar 2021)	
	Number	Incidence per 10,000 OBDs (95%CI)	Number	Incidence per 10,000 OBDs (95%CI)
Bloodstream cultures	1,518	5.3 (5.0-5.6)	470	4.9 (4.5–5.4)
Urinary tract cultures	5,781	20.2 (19.7–20.7)	1,916	20.1 (19.2–21.1)
Total	7,299	25.5 (24.9–26.1)	2,386	25.1 (24.1–26.1)
*Occupied bed days*	*2,864,089*	*--*	*951,042*	--

OBDs – Occupied bed days

95%CI – 95% Confidence intervals

Differences in laboratory reporting nomenclature, and small numbers of certain species, resulted in the grouping of several species for analysis, such as *Escherichia* species, *Staphylococcus* species and *Candida* species (Supplementary Table S2). *Escherichia* species were the most frequently identified organism in both cohorts (Table 2 and Supplementary Tables 3 and 4).

**Table 2:** Frequency of most common organisms by cohort*

Pre-COVID-19 cohort (Jan 2017 – Feb 2020) (n = 6566)			COVID-19 cohort (Mar 2020 – Mar 2021) (n = 3119)		
Organism	Number	Proportion	Organism	Number	Proportion
*Escherichia* species	1746	26.6%	*Escherichia* species	728	23.4%
*Enterococcus* species	1069	16.3%	*Enterococcus* species	427	13.7%
*Candida* species	809	12.3%	*Candida* species	388	12.4%
*Klebsiella* species	523	8.0%	*Klebsiella* species	242	7.8%
*Pseudomonas* species	510	7.8%	*Pseudomonas* species	205	6.6%
*Staphylococcus* species	287	4.4%	*Staphylococcus* species^Ω^	183	5.9%
*Proteus* species	257	3.9%	*Enterobacter* species	141	4.5%
*Enterobacter* species	244	3.7%	VRE	140	4.5%
VRE	188	2.9%	MSSA	100	3.2%
*Citrobacter* species	115	1.7%	*Proteus* species	71	2.3%

*Not all organisms reported (only most common 10 species)

^#^ Includes cultures where more than one organism was reported

^Ω^*Staphylococcus* other than aureus

VRE - Vancomycin resistant *enterococci*

MSSA – methicillin sensitive *Staphylococcus aureus*

### Time series analysis of pre COVID-19 cohort and COVID-19 cohort

#### Combined bloodstream and urinary tract infections

**Fig. 1:**
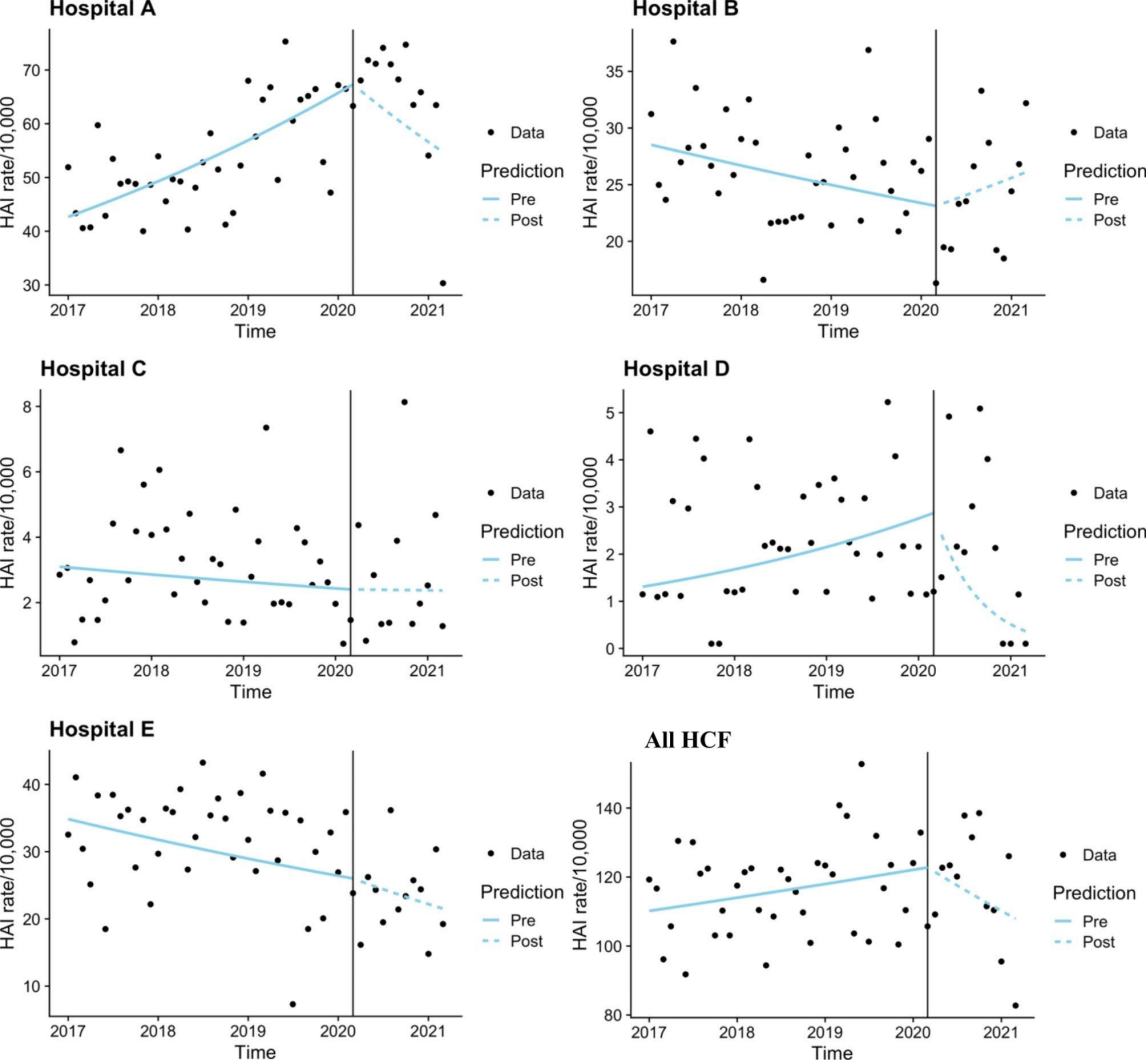
Time series analysis – Combined BSI and UTI by hospital

There was no significant difference in the two cohorts when all hospital data was pooled (Fig. 1). Across all services, the incidence rate of infection was increasing in the pre-COVID-19 cohort (p = 0.077), with a drop of approximately 1 case per month (back-transformed) in the COVID-19 cohort (p = 0.064). Hospital A demonstrated a significant increase in the pre-COVID-19 cohort (p < 0.001), and a significant decrease in the COVID-19 cohort (p = 0.004) when combining both BSI and UTI data. Hospital D had a significant decrease in the COVID-19 cohort (p = 0.002). There were no other significant trends identified, however Hospitals C and D had a slight decrease in the COVID-19 cohort, whilst Hospital B demonstrated an increase in the COVID-19 cohort.

#### Bloodstream infections

**Fig. 2:**
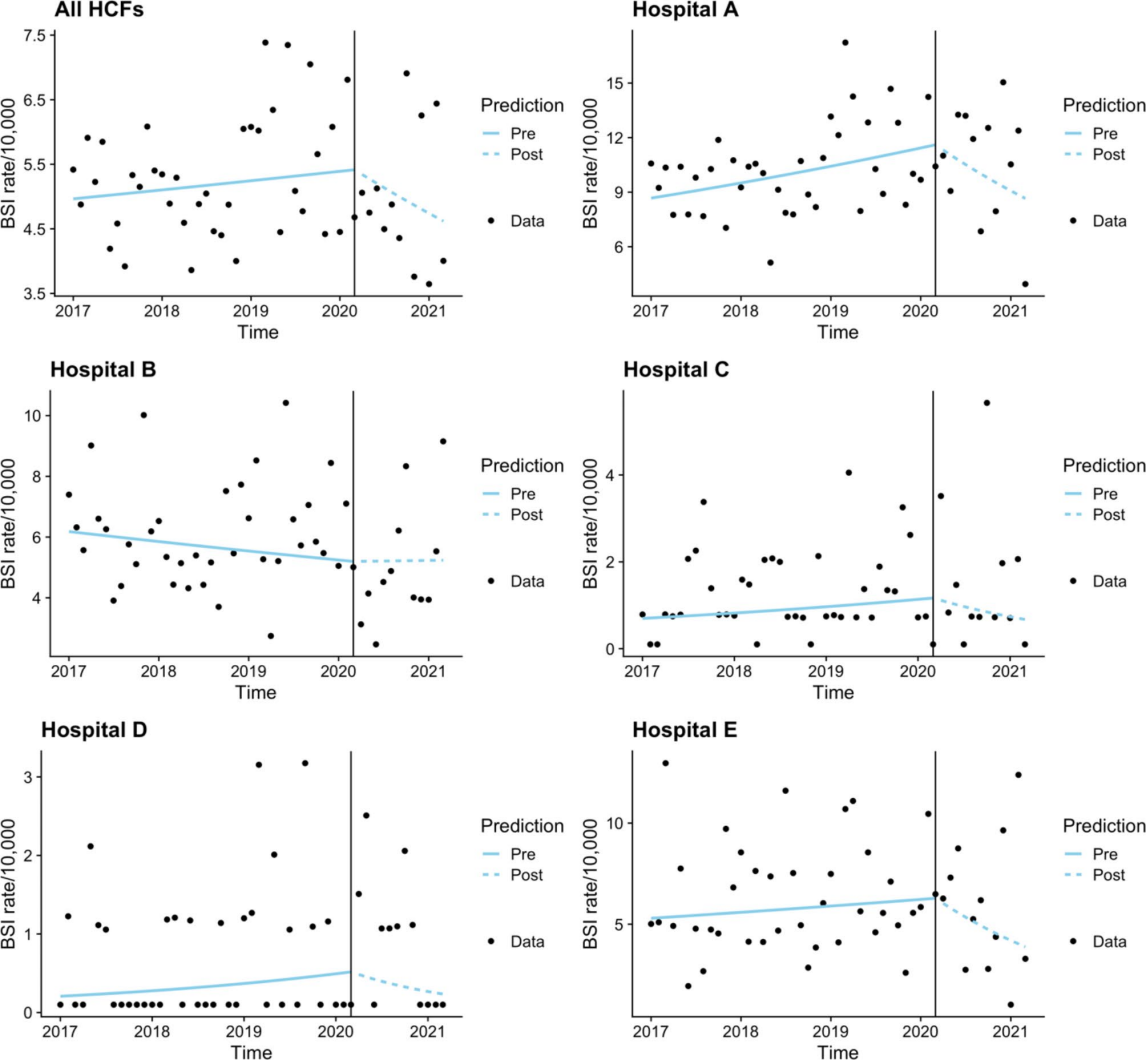
Time series analysis - Bloodstream infections combined and by hospital

When combing all BSI data, although a downward trend is noted in the COVID-19 cohort, it was not significant (Fig. 2). Hospital A had significant increase in BSI in the pre COVID-19 cohort (p = 0.028) and a significant decrease in the COVID-19 cohort (p = 0.042). No other significant trends were identified, however Hospitals C, D and E all had downward trends in the COVID-19 cohort.

#### Urinary tract infections

**Fig. 3:**
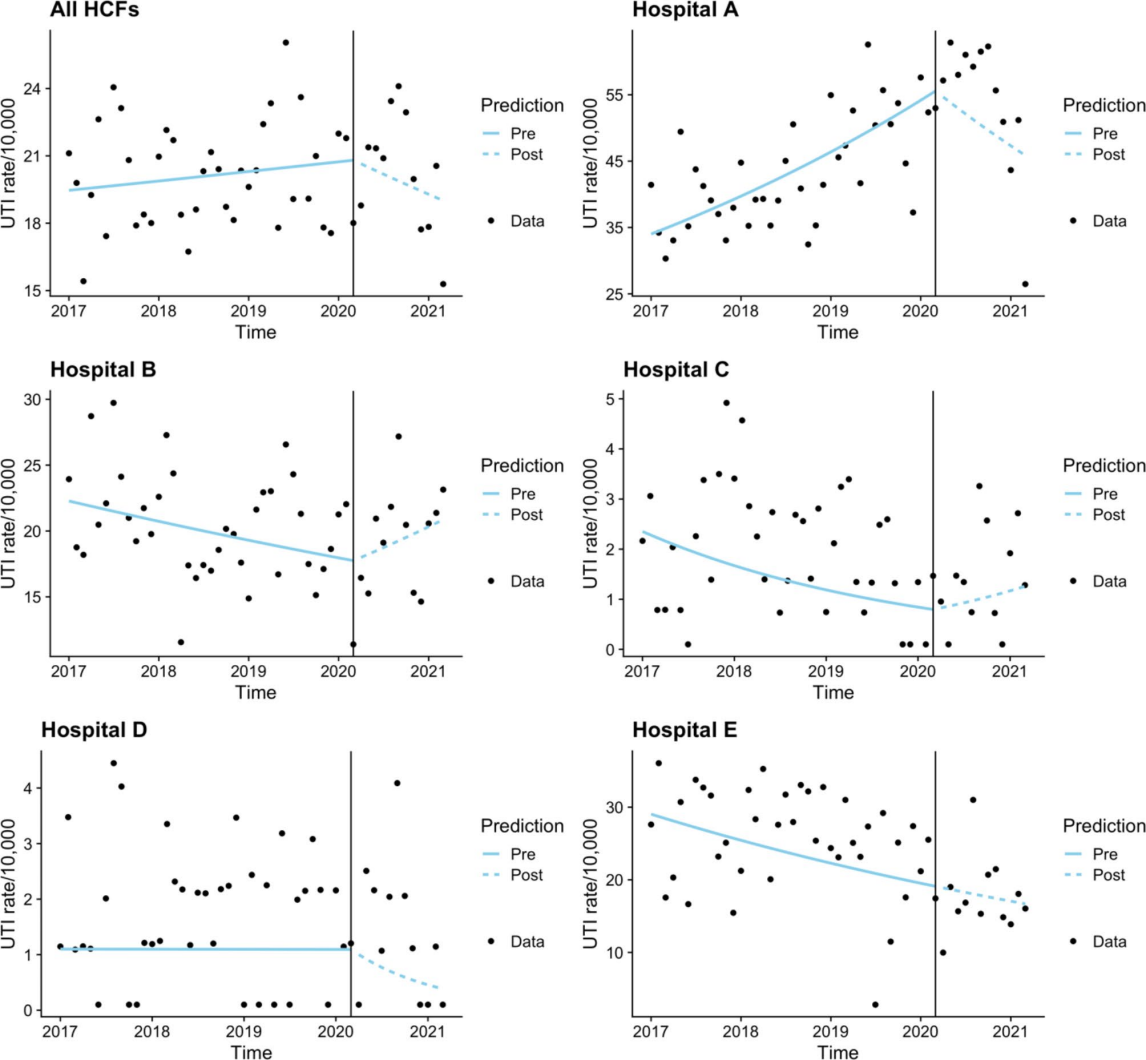
Time series analysis – Urinary tract infections combined and by hospital

There is a downward trend in the COVID-19 cohort when combining all hospitals UTI data, however it was not significant (Fig. 3). Hospital A had a significant increase in the pre-COVID-19 cohort (p < 0.001) and a significant decrease in the COVID-19 cohort (p = 0.005). Hospitals B, C and D all demonstrated significant decreases in UTI in the pre-COVID-19 cohort (p = 0.026, p = 0.043 and p = 0.041 respectively), whilst hospital B and C showed an increase in the COVID-19 cohort, and Hospitals D and E had a downward trend, none were significant.

#### Combined infections by state

**Fig. 4:**
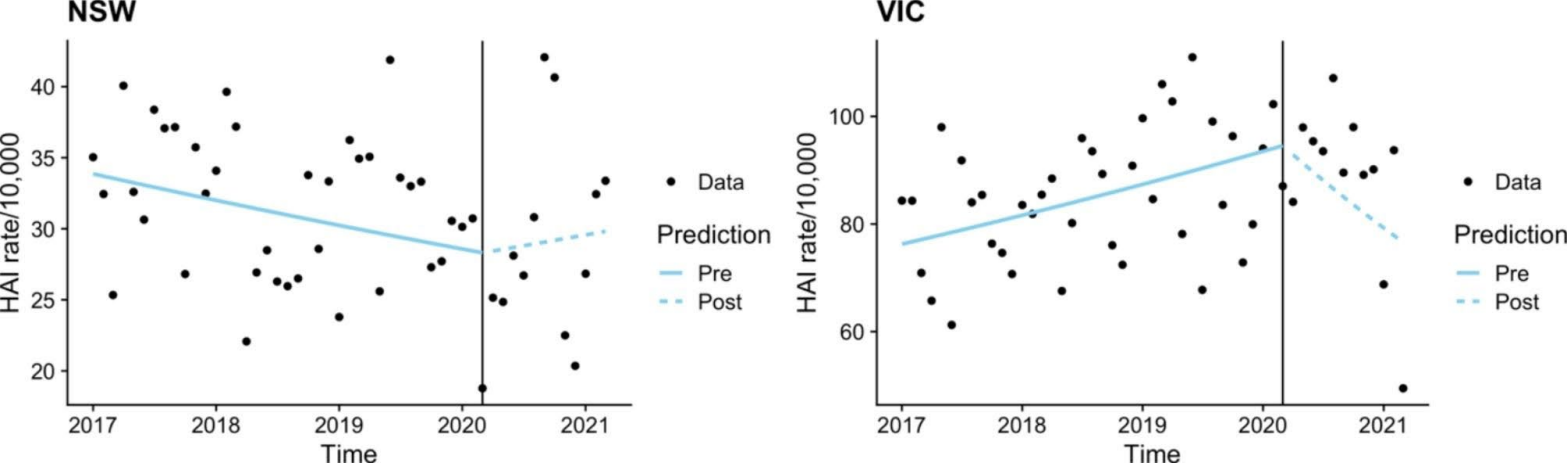
Time series analysis – Bloodstream infections and Urinary tract infections combined by state

Combining BSI and UTI data and grouping by state demonstrated that Victoria had a significant increase in the pre-COVID-19 cohort (p = 0.005) and a significant decrease in the COVID-19 cohort (p = 0.011). No significant trends were identified in combined NSW data (Fig. 4).

## Discussion

This is the first multicentred study exploring the impact of COVID-19 on healthcare associated infections in Australian hospitals using positive blood and urine cultures post 48 h admission as a proxy marker, resulting in mixed findings that may have several explanations.

Australia’s first case of COVID-19 was identified on 25th January 2020 in Victoria. By mid-March, Australia had closed its international borders, and towards the end of March 2020, States and Territories had implemented stay at home orders. By the end of 2020, there were approximately 28,500 cases Australia wide highlighted by two distinct peaks; nationally in March and April, and in Victoria in June to September [22]. There were also differences in the epidemiology between states. In this study, we reviewed data from Victorian and NSW hospitals only.

Victoria experienced Australia’s largest COVID-19 wave in 2020 (Supplementary Figure S3) and implemented enhanced infection prevention measures prior to NSW. This may influence the decrease in HAIs in Victorian hospitals in this data (Hospitals A and E). According to the local epidemiology, hospitals implemented enhanced infection prevention and controls, limitations on visitors and a decrease in elective surgery at various times during the year, largely directed by the local authority. Furthermore, public sector hospitals had a higher burden of COVID-19 patients than the private sector which may have also influenced our data. The inpatient population also changed during 2020. A decrease in elective surgery facilitated the establishment of COVID-19 wards, capacity for intensive care beds increased, and the use of telehealth possibly enabled some patients to remain out of hospital. Staff were redeployed to areas of greatest need, and many staff were furloughed for periods of up to two weeks if they had COVID-19 or were a close contact. Whilst enhanced infection prevention activity may be expected to reduce HAIs, the changes in patient populations and staff profile may in fact increase the risk of HAI.

Our mixed findings reflect the uncertainty of the effect COVID-19 has had on HAIs in other settings. Although there are numerous reports of increases in HAI, [6, 23–30] and decreases, [31–35] variations in settings and methodology prevent comparisons between those findings and with our study.

There are a number of limitations with this study. Without a national HAI surveillance program in Australia, the effect of COVID-19 on HAIs nationally is unable to be estimated. As such, we have used proxy measures of HAI being positive cultures from blood and urine that were sampled greater than 48 h post admission from five hospitals. We did not explore the triggers for taking cultures within each hospitals, therefore our results could have been influenced by differences in the practices of taking cultures between hospitals. However, we expect that practices for taking cultures within each individual hospital would have remained relatively stable during the study period. Although we had data from five hospitals, the number of positive cultures were relatively small. The period of data collection for the COVID-19 cohort was 13 months, which resulted in lower levels of statistical power to detect trends in the second study period compared with the first period. Finally, differences in reporting between the hospital laboratories meant that we had to report some groups at a genus level only, and data were not reviewed for potential contaminants.

## Conclusion

Although the findings of this study are uncertain, such large and widespread increase in the awareness and implementation of infection prevention in hospitals nationally warrant further research. The COVID-19 pandemic has and will continue to have significant impact on healthcare in Australia, whilst much of the response is reactive, we must also continue to explore effectiveness of infection prevention and control measures and adapt as knowledge increases. Further larger studies that aggregate hospitals by state, and by hospital category, with time series analyses performed which consider the local epidemiology of COVID, may provide further insight on the effect of COVID on HAIs.

## Electronic supplementary material

Below is the link to the electronic supplementary material.

Supplementary Material 1

## Data Availability

The datasets used and/or analysed during the current study are available from the corresponding author on reasonable request.

## Abbreviations

BSI: Bloodstream infection
HAI: Healthcare associated infection
IPC: Infection prevention and control
ITS: Interrupted time series
NSW: New South Wales
OBD: Occupied bed days
PPE: Personal protective equipment
UTI: Urinary tract infection
