# Supplementary material for: The incidence of nosocomial bloodstream infection and urinary tract infection in Australian hospitals before and during the COVID-19 pandemic: an interrupted time series study
Source: Antimicrob Resist Infect Control. 2023 Jul 3;12:61. doi: 10.1186/s13756-023-01268-2 (PMC10318831; doi:10.1186/s13756-023-01268-2)
Supplement: Supplementary file 1 — Supplementary Material 1 [file 13756_2023_1268_MOESM1_ESM.docx]

## Supplementary File

**Table S1 - Australian hospital peer groups**

| **Category** | Definition |
| --- | --- |
| **Principal referral hospitals** | Provide a very broad range of services, including some very sophisticated services, and have very large patient volumes. Most include an intensive care unit, a cardiac surgery unit, a neurosurgery unit, an Infectious diseases unit and a 24-hour emergency department. |
| **Public acute group A hospitals** | Provide a wide range of services to a large number of patients and are usually in metropolitan centres or inner regional areas. Most have an intensive care unit and a 24-hour emergency department. They are among the largest hospitals, but provide a narrower range of services than *Principal referral* hospitals. They have a range of specialist units, potentially including bone marrow transplant, coronary care and oncology units. |
| **Private acute group A hospitals** | Private acute hospitals that have a 24-hour emergency  department and an intensive care unit, and provide a number of other specialised services  such as coronary care, special care nursery, cardiac surgery and neurosurgery. |

Australian hospital peer groups. Health services series no. 66. Cat. no. HSE 170. (Canberra: AIHW) (2015).

**Table S2- Pathogen Groups**

| **Organism reported** | **Pathogen Group** |
| --- | --- |
| Abiotrophia species | Abiotrophia species |
| Achromobacter species | Achromobacter species |
| Acinetobacter baumannii complex | Acinetobacter baumannii complex |
| Acinetobacter species | Acinetobacter species |
| Actinomyces neuii | Acinetobacter species |
| Actinomyces species | Acinetobacter species |
| Actinotignum (Actinobaculum) schaalii | Actinotignum species |
| Aerococcus sanguinicola | Aerococcus species |
| Aerococcus urinae | Aerococcus species |
| Aeromonas species | Aeromonas species |
| Aeromonas veronii species | Aeromonas veronii species |
| Aggregatibacter species | Aggregatibacter species |
| Anaerobic gram negative species | Anaerobic gram negative species |
| Anaerobic gram positive species | Anaerobic gram positive species |
| Bacillus species | Bacillus species |
| Bacteroides species | Bacteroides species |
| Bifidobacterium species | Bifidobacterium species |
| Burkholderia cepacia species | Burkholderia cepacia species |
| Burkholderia species | Burkholderia species |
| Burkholderia stabilis | Burkholderia species |
| Campylobacter species | Campylobacter species |
| Candida albicans | Candida species |
| Candida dubliniensis | Candida species |
| Candida glabrata complex | Candida species |
| Candida species | Candida species |
| Candida tropicalis | Candida species |
| Capnocytophaga species | Anaerobic gram negative species |
| Chryseobacterium species | Chryseobacterium species |
| Citrobacter amalonaticus | Citrobacter species |
| Citrobacter farmeri | Citrobacter species |
| Citrobacter freundii | Citrobacter species |
| Citrobacter freundii complex | Citrobacter species |
| Citrobacter koseri | Citrobacter species |
| Citrobacter species | Citrobacter species |
| Citrobacter youngae | Citrobacter species |
| Clavispora (Candida) lusitaniae | Candida species |
| Clostridium species | Clostridium species |
| Corynebacterium aurimucosum | Corynebacterium species |
| Corynebacterium durum | Corynebacterium species |
| Corynebacterium jeikeium | Corynebacterium species |
| Corynebacterium species | Corynebacterium species |
| Cryptococcus species | Cryptococcus species |
| Delftia species | Delftia species |
| Dermacoccus species | Dermacoccus species |
| Elizabethkingia species | Elizabethkingia species |
| Enterobacter cloacae | Enterobacter species |
| Enterobacter cloacae complex | Enterobacter species |
| Enterobacter species | Enterobacter species |
| Enterococcus avium | Enterococcus species |
| Enterococcus casseliflavus | Enterococcus species |
| Enterococcus faecalis | Enterococcus species |
| Enterococcus faecium | Enterococcus species |
| Enterococcus faecium (VRE) | VRE species |
| Enterococcus raffinosus | Enterococcus species |
| Enterococcus species | Enterococcus species |
| ESBL | ESBL |
| Escherichia coli | Escherichia species |
| Escherichia species | Escherichia species |
| Flavonifractor species | Flavonifractor species |
| Fusobacterium species | Fusobacterium species |
| Gemella species | Gemella species |
| Geotrichum species | Geotrichum species |
| Gordonia species | Gordonia species |
| Gram Negative Rod | Other GNR |
| Gram Positive Cocci | Gram positive species |
| Gram positive species | Other GPR |
| Granulicatella species | Granulicatella species |
| Haemophilus parainfluenzae | Haemophilus species |
| Haemophilus species | Haemophilus species |
| Hafnia alvei | Hafnia species |
| Hafnia species | Hafnia species |
| Helicobacter species | Helicobacter species |
| Klebsiella (Enterobacter) aerogenes | Enterobacter species |
| Klebsiella oxytoca | Klebsiella species |
| Klebsiella pneumoniae | Klebsiella species |
| Klebsiella species | Klebsiella species |
| Klebsiella variicola | Klebsiella species |
| Lachnoanaerobaculum species | Lachnoanaerobaculum species |
| Lactobacillus species | Lactobacillus species |
| Leclercia species | Leclercia species |
| Leptotrichia species | Leptotrichia species |
| Leuconostoc species | Leuconostoc species |
| Micrococcus species | Micrococcus species |
| Morganella morganii | Morganella species |
| Morganella species | Morganella species |
| MRSA | MRSA |
| MSSA | MSSA |
| Mycobacterium abscessus species | Mycobacterium abscessus species |
| Mycobacterium avium species | Mycobacterium avium species |
| Mycobacterium bovis | Mycobacterium species |
| Mycobacterium species | Mycobacterium species |
| Mycobacterium tuberculosis | Mycobacterium species |
| Neisseria flavescens species | Neisseria flavescens species |
| Neisseria species | Neisseria species |
| Nocardia species | Nocardia species |
| Other GNR | OTHER GNR |
| Other GPR | OTHER GPR |
| Pantoea species | Pantoea species |
| Parabacteroides species | Parabacteroides species |
| Paracraurococcus species | Paracraurococcus species |
| Parvimonas species | Parvimonas species |
| Peptostreptococcus species | Peptostreptococcus species |
| Pichia (C. krusei) kudriavzevii | Candida species |
| Prevotella species | Prevotella species |
| Proteus mirabilis | Proteus species |
| Proteus penneri | Proteus species |
| Proteus species | Proteus species |
| Proteus vulgaris | Proteus species |
| Providencia rettgeri | Providencia species |
| Pseudomonas aeruginosa | Pseudomonas species |
| Pseudomonas fluorescens | Pseudomonas species |
| Pseudomonas putida | Pseudomonas species |
| Pseudomonas species | Pseudomonas species |
| Raoultella ornithinolytica | Raoultella species |
| Raoultella planticola | Raoultella species |
| Raoultella species | Raoultella species |
| Raoultella terrigena | Raoultella species |
| Rhizopus microsporus | Rhizopus species |
| Ruminococcus species | Ruminococcus species |
| Saccharomyces cerevisiae | Saccharomyces species |
| Saccharomyces species | Saccharomyces species |
| Salmonella species | Salmonella species |
| Scedosporium species | Scedosporium species |
| Serratia marcescens | Serratia species |
| Serratia species | Serratia species |
| Shewanella species | Shewanella species |
| Sphingomonas species | Sphingomonas species |
| Staphylococcus aureus | MSSA |
| Staphylococcus aureus (MRSA) | MRSA |
| Staphylococcus capitis | MSSA |
| Staphylococcus epidermidis | Staphylococcus species |
| Staphylococcus haemolyticus | Staphylococcus species |
| Staphylococcus hominis | Staphylococcus species |
| Staphylococcus lugdunensis | Staphylococcus species |
| Staphylococcus saprophyticus | Staphylococcus species |
| Staphylococcus simulans | Staphylococcus species |
| Staphylococcus species | Staphylococcus species |
| Staphylococcus warneri | Staphylococcus species |
| Stenotrophomonas maltophilia | Stenotrophomonas species |
| Stenotrophomonas species | Stenotrophomonas species |
| Streptococcus agalactiae (Group B) | Streptococcus species |
| Streptococcus anginosus group | Streptococcus species |
| Streptococcus dysgalactiae | Streptococcus species |
| Streptococcus gallolyticus | Streptococcus species |
| Streptococcus infantarius | Streptococcus species |
| Streptococcus mitis group | Streptococcus species |
| Streptococcus pyogenes (Group A) | Streptococcus species |
| Streptococcus species | Streptococcus species |
| Trichosporon asahii | Trichosporon species |
| Trichosporon species | Trichosporon species |
| Ustilago species | Trichosporon species |
| Veillonella species | Veillonella species |
| VRE | VRE species |
| Weissella species | Weissella species |
| Yeast | Candida species |
| Yersinia species | Yersinia species |

**Table S3 – Frequency of organisms in the Pre-COVID-19 cohort (Jan 2017 – Feb 2020) n=6566**

| **Organism** | **Number** | **Proportion** |
| --- | --- | --- |
| *Escherichia* species | 1746 | 26.6% |
| *Enterococcus* species | 1069 | 16.3% |
| *Candida* species | 809 | 12.3% |
| *Klebsiella* species | 523 | 8.0% |
| *Pseudomonas* species | 510 | 7.8% |
| *Staphylococcus* species | 287 | 4.4% |
| *Proteus* species | 257 | 3.9% |
| *Enterobacter* species | 244 | 3.7% |
| VRE | 188 | 2.9% |
| *Citrobacter* species | 115 | 1.7% |
| MSSA | 111 | 1.7% |
| *Streptococcus* species | 100 | 1.5% |
| ESBL | 87 | 1.3% |
| *Morganella* species | 67 | 1.0% |
| *Serratia* species | 67 | 1.0% |
| MRSA | 43 | 0.7% |
| *Bacteroides* species | 32 | 0.5% |
| Other gram negative rods | 31 | 0.5% |
| *Helicobacter* species | 30 | 0.5% |
| *Stenotrophomonas* species | 27 | 0.4% |
| Gram positive species | 24 | 0.4% |
| *Acinetobacter baumannii* complex | 15 | 0.2% |
| *Actinomyces* species | 15 | 0.2% |
| *Acinetobacter* species | 14 | 0.2% |
| *Clostridium* species | 12 | 0.2% |
| *Raoultella* species | 11 | 0.2% |
| *Hafnia* species | 10 | 0.2% |
| *Elizabethkingia* species | 9 | 0.1% |
| *Burkholderia* species | 6 | 0.1% |
| *Haemophilus* species | 6 | 0.1% |
| *Fusobacterium* species | 6 | 0.1% |
| *Salmonella* species | 6 | 0.1% |
| *Peptostreptococcus* species | 5 | 0.1% |
| *Prevotella* species | 5 | 0.1% |
| *Saccharomyces* species | 4 | 0.1% |
| *Veillonella* species | 4 | 0.1% |
| *Corynebacterium* species | 3 | 0.0% |
| *Lactobacillus* species | 3 | 0.0% |
| Other gram positive rods | 3 | 0.0% |
| *Weissella* species | 3 | 0.0% |
| *Abiotrophia* species | 2 | 0.0% |
| *Aeromonas veronii* species | 2 | 0.0% |
| Anaerobic gram negative species | 2 | 0.0% |
| Anaerobic gram positive species | 2 | 0.0% |
| *Bacillus* species | 2 | 0.0% |
| *Gemella* species | 2 | 0.0% |
| *Granulicatella* species | 2 | 0.0% |
| *Leptotrichia* species | 2 | 0.0% |
| *Micrococcus* species | 2 | 0.0% |
| *Mycobacterium* *avium* species | 2 | 0.0% |
| *Neisseria* species | 2 | 0.0% |
| *Achromobacter* species | 2 | 0.0% |
| *Burkholderia* cepacia species | 2 | 0.0% |
| *Campylobacter* species | 2 | 0.0% |
| *Cryptococcus* species | 2 | 0.0% |
| *Dermacoccus* species | 2 | 0.0% |
| *Gordonia* species | 2 | 0.0% |
| *Lachnoanaerobaculum* species | 2 | 0.0% |
| *Leclercia* species | 2 | 0.0% |
| *Sphingomonas* species | 2 | 0.0% |
| *Aeromonas* species | 1 | 0.0% |
| *Aggregatibacter* species | 1 | 0.0% |
| *Chryseobacterium* species | 1 | 0.0% |
| *Delftia* species | 1 | 0.0% |
| *Finegoldia* species | 1 | 0.0% |
| *Flavonifractor* species | 1 | 0.0% |
| *Leuconostoc* species | 1 | 0.0% |
| *Mycobacterium abscessus* species | 1 | 0.0% |
| *Mycobacterium* species | 1 | 0.0% |
| *Neisseria flavescens* species | 1 | 0.0% |
| *Nocardia* species | 1 | 0.0% |
| *Pantoea* species | 1 | 0.0% |
| *Parabacteroides* species | 1 | 0.0% |
| *Paracraurococcus* species | 1 | 0.0% |
| *Parvimonas* species | 1 | 0.0% |
| *Ruminococcus* species | 1 | 0.0% |
| *Shewanella* species | 1 | 0.0% |

**Table S4 – Frequency of organisms in the COVID-19 cohort (Mar 2020 – Mar 2021) n=3119**

| **Organism** | **Number** | **Proportion** |
| --- | --- | --- |
| *Escherichia* species | 728 | 23.4% |
| *Enterococcus* species | 427 | 13.7% |
| *Candida* species | 388 | 12.4% |
| *Klebsiella* species | 242 | 7.8% |
| *Pseudomonas* species | 205 | 6.6% |
| *Staphylococcus* species | 183 | 5.9% |
| *Enterobacter* species | 141 | 4.5% |
| VRE | 140 | 4.5% |
| MSSA | 100 | 3.2% |
| *Proteus* species | 71 | 2.3% |
| *Streptococcus* species | 67 | 2.1% |
| *Serratia* species | 50 | 1.6% |
| *Citrobacter* species | 40 | 1.3% |
| MRSA | 38 | 1.2% |
| ESBL | 33 | 1.1% |
| *Bacteroides* species | 31 | 1.0% |
| *Morganella* species | 30 | 1.0% |
| *Stenotrophomonas* species | 26 | 0.8% |
| *Helicobacter* species | 16 | 0.5% |
| *Acinetobacter baumannii* complex | 12 | 0.4% |
| Other gram negative rods | 12 | 0.4% |
| *Clostridium* species | 10 | 0.3% |
| *Raoultella* species | 9 | 0.3% |
| *Acinetobacter* species | 8 | 0.3% |
| Gram positive species | 8 | 0.3% |
| *Actinomyces* species | 7 | 0.2% |
| *Fusobacterium* species | 6 | 0.2% |
| *Haemophilus* species | 6 | 0.2% |
| *Prevotella* species | 6 | 0.2% |
| *Salmonella* species | 4 | 0.1% |
| *Burkholderia* species | 3 | 0.1% |
| *Corynebacterium* species | 3 | 0.1% |
| Other gram positive rods | 3 | 0.1% |
| *Abiotrophia* species | 2 | 0.1% |
| Anaerobic gram positive species | 2 | 0.1% |
| *Bacillus* species | 2 | 0.1% |
| *Burkholderia cepacia* species | 2 | 0.1% |
| *Granulicatella* species | 2 | 0.1% |
| *Lachnoanaerobaculum* species | 2 | 0.1% |
| *Lactobacillus* species | 2 | 0.1% |
| *Leptotrichia* species | 2 | 0.1% |
| *Micrococcus* species | 2 | 0.1% |
| *Neisseria* species | 2 | 0.1% |
| *Peptostreptococcus* species | 2 | 0.1% |
| *Saccharomyces* species | 2 | 0.1% |
| *Achromobacter* species | 2 | 0.1% |
| Anaerobic gram negative species | 2 | 0.1% |
| *Campylobacter* species | 2 | 0.1% |
| *Cryptococcus* species | 2 | 0.1% |
| *Dermacoccus* species | 2 | 0.1% |
| *Elizabethkingia* species | 2 | 0.1% |
| *Gordonia* species | 2 | 0.1% |
| *Hafnia* species | 2 | 0.1% |
| *Mycobacterium avium* species | 2 | 0.1% |
| *Mycobacterium* species | 2 | 0.1% |
| *Sphingomonas* species | 2 | 0.1% |
| *Trichosporon* species | 2 | 0.1% |
| *Veillonella* species | 2 | 0.1% |
| *Aeromonas* species | 1 | 0.0% |
| *Aeromonas veronii* species | 1 | 0.0% |
| *Aggregatibacter* species | 1 | 0.0% |
| *Chryseobacterium* species | 1 | 0.0% |
| *Delftia* species | 1 | 0.0% |
| *Finegoldia* species | 1 | 0.0% |
| *Flavonifractor* species | 1 | 0.0% |
| *Gemella* species | 1 | 0.0% |
| *Leclercia* species | 1 | 0.0% |
| *Leuconostoc* species | 1 | 0.0% |
| *Mycobacterium abscessus* species | 1 | 0.0% |
| *Neisseria flavescens* species | 1 | 0.0% |
| *Nocardia* species | 1 | 0.0% |
| *Pantoea* species | 1 | 0.0% |
| *Parabacteroides* species | 1 | 0.0% |
| *Paracraurococcus* species | 1 | 0.0% |
| *Parvimonas* species | 1 | 0.0% |
| *Ruminococcus* species | 1 | 0.0% |
| *Shewanella* species | 1 | 0.0% |
| *Weissella* species | 1 | 0.0% |

**Figure S1 - Comparison of occupied bed days by month**

**Figure S2: Jack-knife sensitivity analysis**


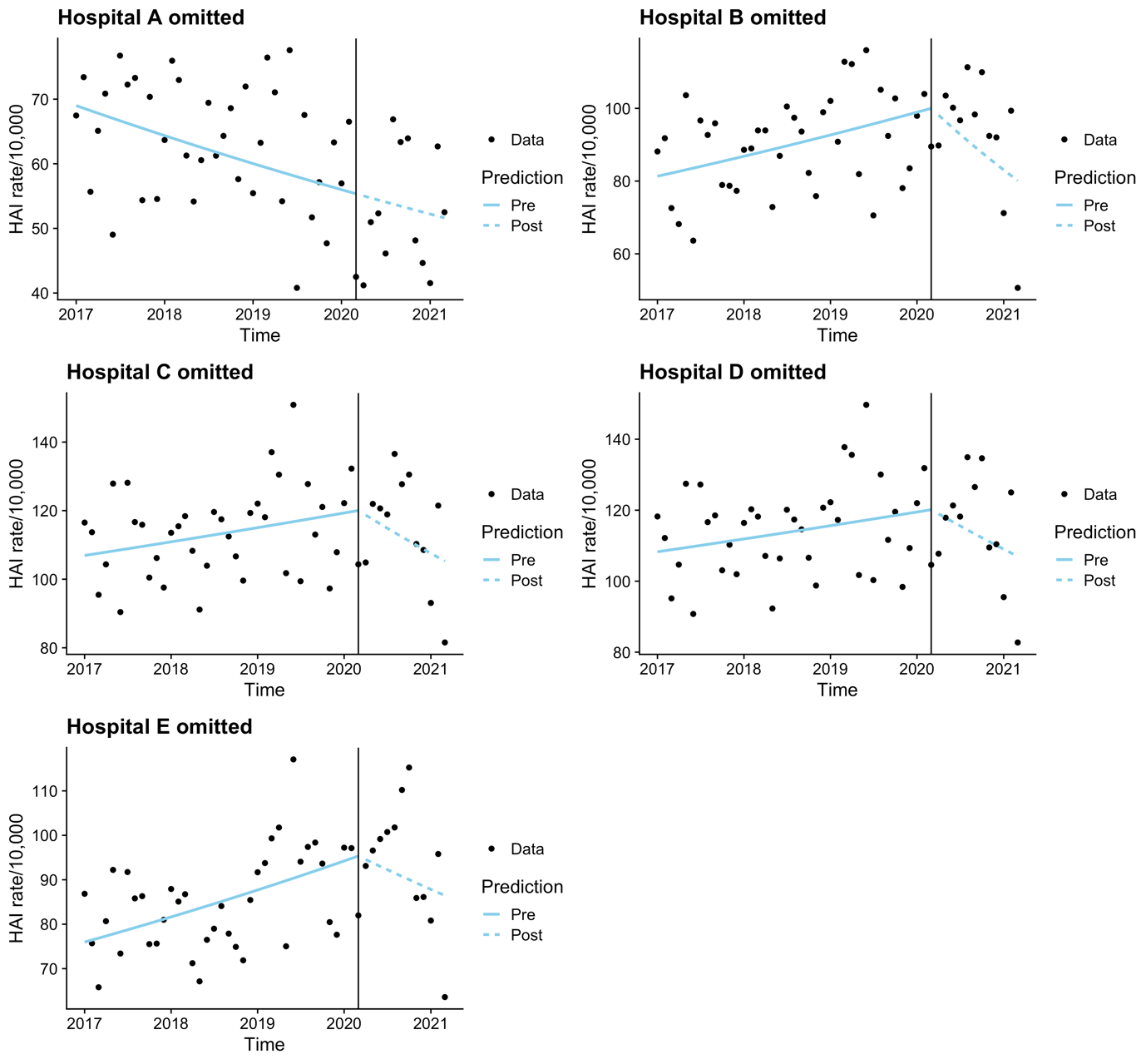


**Figure S3: New COVID-19 cases and COVID-19 hospitalisations in Victoria and NSW**
